# Structure-guided insights into potential function of novel genetic variants in the malaria vaccine candidate PfRh5

**DOI:** 10.1038/s41598-022-23929-9

**Published:** 2022-11-12

**Authors:** Khadidiatou Mangou, Adam J. Moore, Laty Gaye Thiam, Aboubacar Ba, Alessandra Orfanó, Ife Desamours, Duncan Ndungu Ndegwa, Justin Goodwin, Yicheng Guo, Zizhang Sheng, Saurabh D. Patel, Fatoumata Diallo, Seynabou D. Sene, Mariama N. Pouye, Awa Thioub Faye, Alassane Thiam, Vanessa Nunez, Cheikh Tidiane Diagne, Bacary Djilocalisse Sadio, Lawrence Shapiro, Ousmane Faye, Alassane Mbengue, Amy K. Bei

**Affiliations:** 1 Department of Epidemiology of Microbial Diseases, Yale School of Public Health, New Haven, CT USA; 2 G4 - Malaria Experimental Genetic Approaches & Vaccines, Pôle Immunophysiopathologie et Maladies Infectieuses, Institut Pasteur de Dakar, Dakar, Senegal; 3 University of Embu, Embu, Kenya; 4 Zuckerman Mind Brain Behavior Institute, Columbia University, New York, NY USA; 5 Aaron Diamond AIDS Research Center, Columbia University Vagelos College of Physicians and Surgeons, New York, NY USA; 6 Pôle Virologie, Institut Pasteur de Dakar, Dakar, Senegal; 7 Department of Biochemistry and Biophysics, Columbia University, New York, NY USA; 8 Department of Pathology, Microbiology, and Immunology, School of Veterinary Medicine, University of California Davis, Davis, CA USA; 9 MIVEGEC (Infectious Diseases and Vector: Ecology, Genetics, Evolution and Control), University of Montpelier, IRD, CNRS, Montpellier, France

**Keywords:** Evolutionary genetics, Malaria, Molecular modelling

## Abstract

The recent stall in the global reduction of malaria deaths has made the development of a highly effective vaccine essential. A major challenge to developing an efficacious vaccine is the extensive diversity of *Plasmodium falciparum* antigens. While genetic diversity plays a major role in immune evasion and is a barrier to the development of both natural and vaccine-induced protective immunity, it has been under-prioritized in the evaluation of malaria vaccine candidates. This study uses genomic approaches to evaluate genetic diversity in next generation malaria vaccine candidate PfRh5. We used targeted deep amplicon sequencing to identify non-synonymous Single Nucleotide Polymorphisms (SNPs) in PfRh5 (Reticulocyte-Binding Protein Homologue 5) in 189 *P. falciparum* positive samples from Southern Senegal and identified 74 novel SNPs. We evaluated the population prevalence of these SNPs as well as the frequency in individual samples and found that only a single SNP, C203Y, was present at every site. Many SNPs were unique to the individual sampled, with over 90% of SNPs being found in just one infected individual. In addition to population prevalence, we assessed individual level SNP frequencies which revealed that some SNPs were dominant (frequency of greater than 25% in a polygenomic sample) whereas most were rare, present at 2% or less of total reads mapped to the reference at the given position. Structural modeling uncovered 3 novel SNPs occurring under epitopes bound by inhibitory monoclonal antibodies, potentially impacting immune evasion, while other SNPs were predicted to impact PfRh5 structure or interactions with the receptor or binding partners. Our data demonstrate that PfRh5 exhibits greater genetic diversity than previously described, with the caveat that most of the uncovered SNPs are at a low overall frequency in the individual and prevalence in the population. The structural studies reveal that novel SNPs could have functional implications on PfRh5 receptor binding, complex formation, or immune evasion, supporting continued efforts to validate PfRh5 as an effective malaria vaccine target and development of a PfRh5 vaccine.

## Introduction

Malaria remains one of the most prevalent and deadly diseases in the world; responsible for an estimated 241 million cases of disease and 627,000 deaths annually^1^. Over 95% of these cases and deaths are attributed to the parasite *Plasmodium falciparum*. While the field has made great strides in malaria control continued advancement has been near a standstill in recent years^1^ and has even reversed course. A key advancement in *P. falciparum* control came about with the development and licensure of the RTS,S vaccine; the first malaria vaccine, and first human parasite vaccine, to have ever successfully completed Phase III clinical trials and been licensed for widespread use^2^. In the Fall of 2021, the World Health Organization officially recommended the RTS,S vaccine, for children in areas with moderate to high transmission. However, while a tremendous achievement for the field, the RTS,S vaccine shows modest efficacy that wanes with time^3,4^. One of the challenges faced by the RTS,S vaccine is differential efficacy associated with genetic diversity in the target antigen^5^. However, RTS,S is not alone in that strain-specific immunity has eliminated several other malaria vaccine candidates after Phase IIb trails^6–9^. For next generation malaria vaccine development to succeed, a new paradigm is needed that considers natural genetic and phenotypic variation earlier in the vaccine development process, before costly Phase II and III trials.

The highly complex life cycle of *P. falciparum* presents a number of potential stages to which a vaccine can be designed^10^. The human blood-stage infection presents an Achilles’ heel in that successful invasion of human erythrocytes is essential for the completion of the parasite’s life cycle in the human host, and ultimately, transmission to the mosquito^11^. Due to the crucial nature of this life stage, the parasite has expanded invasion ligand families, which are both polymorphic and variantly expressed, that it can deploy to successfully invade the erythrocyte^12^. Among these ligands is one that has been shown to be essential, and thus an attractive vaccine candidate: *Plasmodium falciparum* reticulocyte binding protein homologous 5 (PfRh5)^13^. PfRh5 binds to the Basigin (BSG) receptor on the erythrocyte, marking a crucial and irreversible step in the invasion process^14^. Antibodies targeting the PfRh5-BSG invasion pathway, both on the parasite (PfRh5) and human (BSG) side, have been shown to substantially inhibit invasion efficiency^13,15^. Additionally, a PfRh5 vaccine has induced neutralizing antibodies in *Aoutus* monkeys that are strain-transcendent when challenged with genetically diverse *P. falciparum* isolates in vivo^16,17^.

The genetic diversity that has thwarted the efficacy of previous malaria vaccines is traditionally not specifically evaluated until well into clinical trials^2^. Previous research has shown PfRh5 to be relatively conserved, with 43 non-synonymous polymorphisms (NS SNPs) documented between published and unpublished data^18–23^. Of these documented SNPs, only five have been shown to be present at a prevalence above 10% in any given population, and only one (S197Y) has been shown to contribute to immune evasion from a neutralizing monoclonal antibody^24,25^. However, the sampling for this data is largely taken from lab strains and field isolates that have been adapted to *in vitro* culture. To get ahead of the curve and determine whether a PfRh5 vaccine is likely to face the challenges of natural selection, more investigation and characterization of PfRh5 genetic diversity must be done in highly endemic regions, where genetic diversity and novel SNPs are more likely to naturally occur. In this work, we seek to do just that. We sampled from active cases in Kédougou, Senegal, a highly endemic region already known to contain unique genotypes^20^.

## Results

### Characteristics of study participants

**Figure 1:**
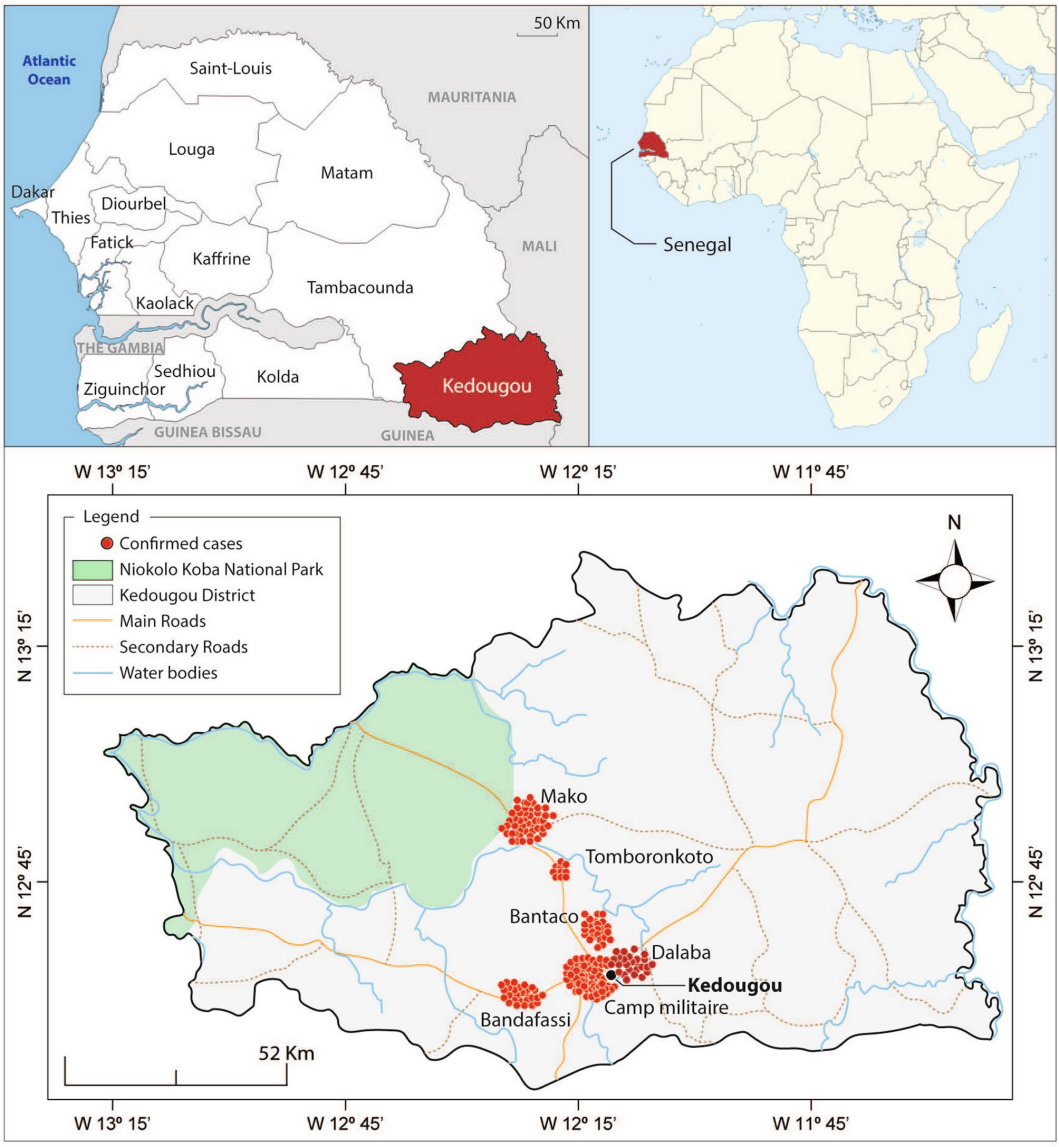
Map of the Study Population: diverse malaria epidemiology within Kédougou Top panel:Kédougou is a region in the South East of Senegal with season malaria transmission from June to December. Bottom panel: The the six sites of enrollment in the present study: Bandafassi, Bantaco, Camp Militaire, Dalaba, Mako, and Tomboronkoto. Each site has a unique epidemiology ranging from rural village (Bandafassi), to mining proximal (Bantaco), to peri-urban (Mako, Tomboronkoto, Dalaba, Camp Militaire). Maps were created with ArcGIS Desktop v10.8.1. (ESRI 2021, Redlands CA; https://desktop.arcgis.com/en/). Red dots represent individual samples enrolled at each site and mapped according to the GPS coordinates of the clinic.

Samples used in this study were collected from Kédougou (Fig. 1) in the Southeastern region of Senegal, with a high seasonal malaria transmission from May to November, annually. The dominant malaria vectors in this region are *Anopheles gambiae s.l.*, *Anopheles funestus*, *Anopheles arabiensis*, and *Anopheles nili*^26^. Participants for this study (N = 189) were individuals aged between 2 to 72 years, presenting symptomatic *P. falciparum* infection, recruited from Bandafassi (25), Bantaco (24), Camp Militaire (63), Dalaba (24), Mako (43) and Tomboronkoto (10) (Fig. 1B). The demographic and parasitological characteristics of the study participants are summarized in Table 1. There was no significant difference observed in the mean ages between the four sampling sites (ANOVA, p = 0.2131), while a significant difference was observed concerning the sex ratio across sites (Chi-square, p = 0.0055). Taken individually, the sex ratio was neutral in Mako and Dalaba; while that of Bantaco and Camp Militaire was in favor of males (3.8 and 2.7, respectively), and the opposite trend was observed in Bandafassi and Mako (0.8 each). No significant difference was observed in the complexity of infection (COI) across sites, while the overall COI was 4.59.

**Table 1:** Patient demographic data and complexity of Infection

Sites	BF	BC	CM	DB	MK	TM	Total	*P*-value^a^
Patients, no.	25	24	63	24	43	10	189	
Sex ratio (male/female)	0.8	3.8	2.7	1	0.8	1	1.46	0.0055^a^
Age (mean, years)	17.96	16.54	21.27	18.57	18.83	26.40	17.76	
[Min–max]	[2–38]	[2–31]	[2–72]	[8–31]	[5–45]	[12–43]	[2–72]	0.1732^b^
COI (mean)	5.56	5.53	4.98	4.31	4.46	4.67	4.59	
[Min–max]	[1–11]	[1–15]	[1–12]	[1–10]	[1–12]	[3–5]	[1–15]	0.0674^b^

^a^Indicates test performed using Chi-square. ^b^Indicates analysis performed with One-way ANOVA. *BF* Bandafassi, *BC* Bantaco, *CM* Camp Millitaire, *DB* Dalaba, *MK* Mako, *TM* Tomboronkoto, *COI* Complexity of Infection.

### Overall prevalence of known and novel SNPs in Kédougou

**Figure 2:**
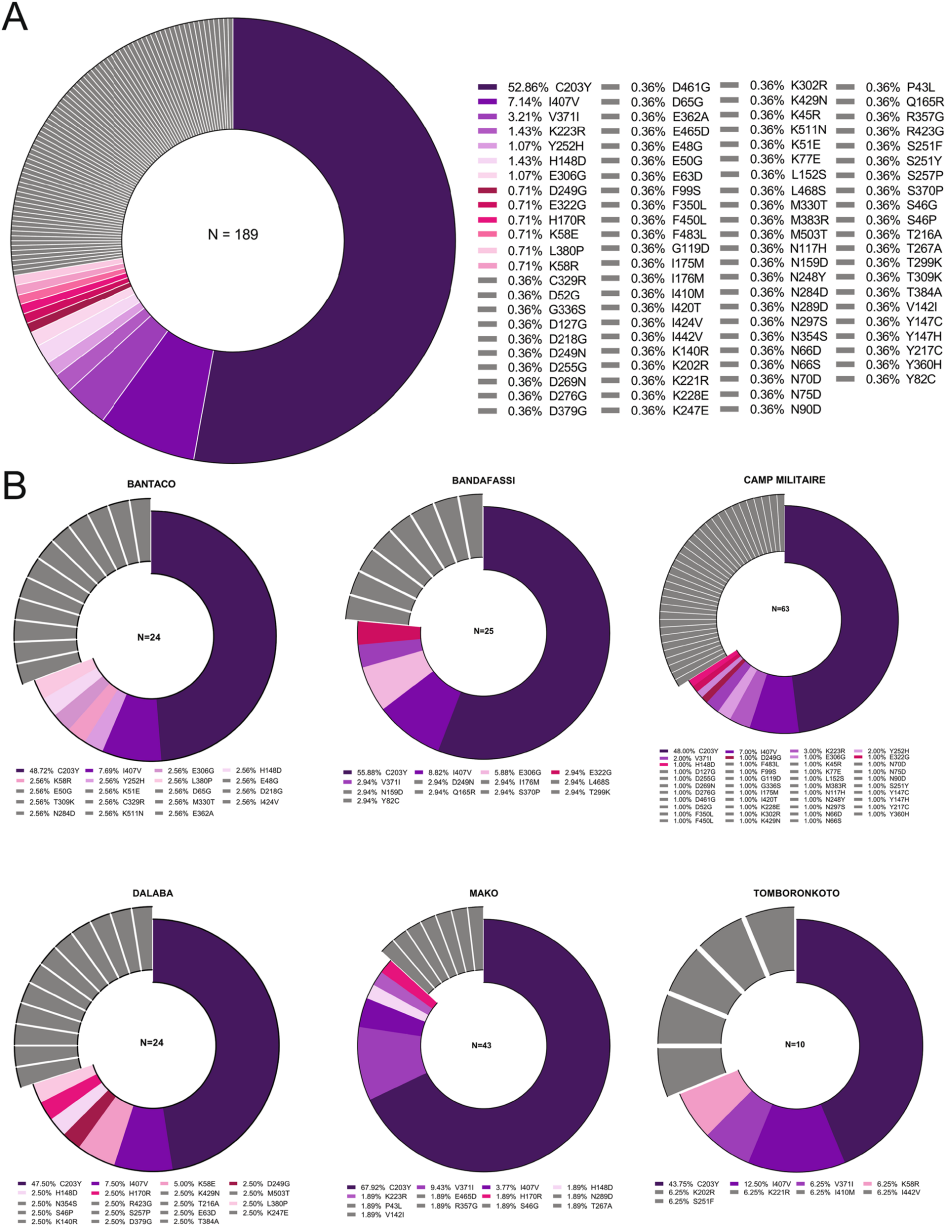
Population prevalence of known and novel SNPs in PfRh5. The prevalence of PfRh5-associated SNPs was calculated as the percentage of SNPs detected within the total sample population (**A**) or within site-specific sample populations (**B**) in Kédougou. The most prevalent SNPs, shared between two or more sites are depicted in degraded colors, while SNPs detected in individual isolates are depicted in grey (**A**). Exploded slices in (**B**) represent SNPs unique to the respective site, while the shared SNPs are shown in matched colors.

We undertook a population-based genetic diversity using deep amplicon sequencing data of PfRh5 obtained from 189 *P. falciparum* clinical samples using Illumina next generation sequencing. We used a very sensitive cut-off (1% variant frequency) to allow maximum SNP discovery, while applying quantitative and qualitative metrics to ensure SNP validity. Overall, 158/189 (83.59%) of the isolates contained at least one mutant allele of PfRh5, relative to the 3D7 reference genome (Fig. 2A, Supplementary Fig. 1). In total, 90 non-synonymous SNPs were identified from these sequences, of which, only 8 were previously reported in other studies^18,21–23^, and an additional 8 were identified in our previously published data^20^. Of the 74 novel SNPs reported here for the first time, 6 were previously described at the same position but we observed different amino acid substitutions (D52G, D127G, D379G, E362A, T384A, and Y147C) (Fig. 2A); Supplementary Table 1). We include the 31 samples in our previously published genotype-phenotype association study^20^ among the 189 total here to allow for a more complete assessment of population prevalence of SNPs in PfRh5. Of the 90 SNPs reported here, 8 were present in more than one isolate, while the bulk 82/90 (91%) were singletons (detected in only individual samples) (Fig. 1). The majority of the SNPs reported in this study were rare variants, as only one was detected at a prevalence greater than 3%, while only two reached over 5% prevalence (C203Y, 52.86% and I407V, 7.14%). Of the novel SNPs detected in the study, only four reach a prevalence of at least 1% in the total population—H148R (1.43%); K223R (1.43%); E306G (1.07%); and Y252H (1.07%)—and six SNPs were detected at a prevalence of 0.71% (D249G, E322G, H170R, K58E, K58R and L380P), while the remaining SNPs were all detected at a prevalence of 0.36%, corresponding to a single sample (Fig. 2A).

### Site specific prevalence of known and novel SNPs in Kédougou

The prevalence of identified SNPs were estimated for each sampling site. As seen in the overall parasite population, the bulk of the isolates reported in the present study presented a mutant allele of PfRh5. By contrast, PfRh5 amplified from 3D7 genomic DNA was included as a control for potential PCR and sequencing errors, and yet 3D7 sequences contained no SNPs. Bantaco presented the highest number of mutant alleles (91.67%) while Bandafassi, Camp Militaire, Mako and Tomboronkoto presented relatively similar prevalences of mutant alleles (80%, 80.95%, 79.17%, 83.72% and 80%, respectively) (Supplementary Fig. 1B–G). Across sites, Camp Militaire presented the highest number of individual SNPs (43 SNPs), followed by Bantaco and Dalaba (19 SNPs each), while samples from Bandafassi and Mako harboured the same number of individual SNPs (13 SNPs each), and the fewest SNPs were detected in isolates from Tomboronkoto (9 SNPs). Interestingly, the bulk of the SNPs detected were unique to the individual sampling sites (not detected at another site). All SNPs unique to a site were additionally only found in a single individual. Isolates from Camp Militaire carrying the highest number of unique SNPs (34/43; 79.06%), followed by Dalaba (12/18; 66.67%), Bantaco (12/19; 63.16%) and Bandafassi (8/13; 61.54%), while the least number of unique SNPs was detected in Mako (7/13; 53.85%) and Tomboronkoto (5/9; 55.56%), roughly proportional to the overall sample numbers from each site (Fig. 2B). Of the remaining SNPs, only C203Y was shared across all sites, while the I407V and V371I mutations were shared in all but one site; I407V was absent from Mako, while V371I was absent from Dalaba. The H148R mutation was observed in isolates from Bantaco, Camp Militaire, Dalaba and Mako, while E306G was only common in isolates from Bantaco, Bandafassi and Camp Militaire. Some SNPs (K58R, H170R, D249G, Y252H, E322G and L380P) were only shared between two sites (Fig. 2B).

### Frequency of novel SNPs in individual samples

**Figure 3:**
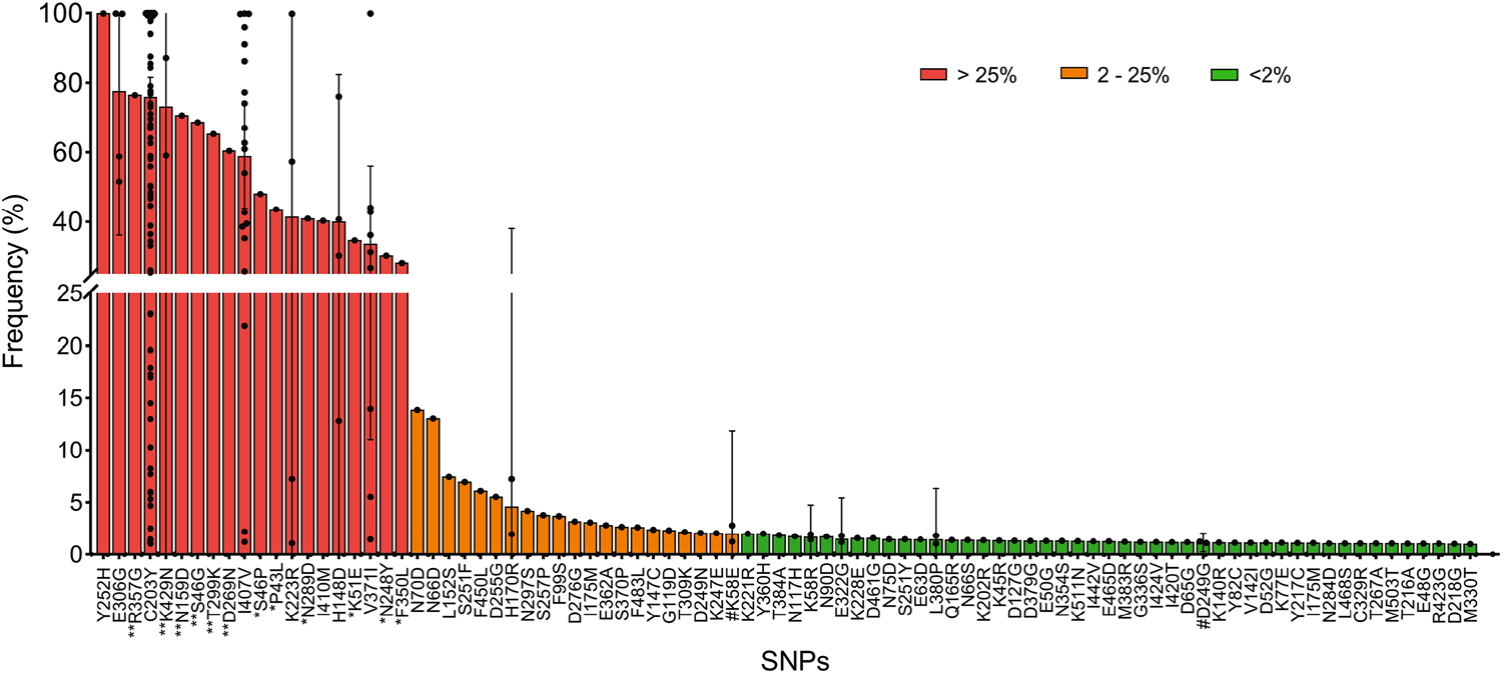
Frequency of PfRh5 SNPs in individual complex infections SNP frequency was determined from the sequencing read data and calculated as the percentage of the variant read coverage relative to the coverage at the variant position. Using quantitative read coverage, we are able to calculate frequency within the overall *Plasmodium* genetic material in a given sample, incorporating multiple genotypes as well as variable parasitemia across genotypes. SNPs are categorized as low < 2% (green), intermediate 2–25% (orange) or high frequency SNPs > 25% (red), based on their respective frequency with the sample population. Additional symbols are used to highlight SNPs occurring at high prevalence but low frequency (“#”), SNPs occurring at low prevalence relatively high (*) or very high frequency (**).

The sequence reads from next generation sequencing experiments were used to assess the frequency of the individual SNPs detected in this study within an individual sample. As the bulk of the isolates analyzed here are mixed genotypes (Average COI 4.59, ranging from 1 to 15 genotypes per sample (Table 1). Due to this high degree of polygenomic infections, and the difficulty with disentangling individual genomes as well as the corresponding parasite burden or parasitemia of each genome in a sample, we calculated the frequency of a variant (SNP) for each isolate based on the overall variant read frequency among reads from individual samples. This allows us to determine the percentage of variant reads among the total at a position and to average across all complex polygenomic infections in the population. Upon analysis, the individual SNPs were categorized based on read frequency within individual samples into low frequency (< 2%), intermediate frequency (2–25%) and high frequency (> 25%) SNPs (Fig. 3). Most SNPs detected at high frequencies in individual isolates were also found at a high population prevalence. A few SNPs were identified at a high frequency in individual samples, but a relatively low population prevalence (in all cases observed in a single sample): F350L, N248Y, K51E, N289D, P43L and S46P with frequencies ranging from 28.1 to 47.98%; and D269N, T299K, S46G, N159D, K429N and R357G with high frequencies ranging from 60.41 to 76.37%. The majority of the novel SNPs described here are present at low to intermediate frequency within a sample and rare in the population (Fig. 3).

### Structure-based insights on potential SNP function

**Figure 4:**
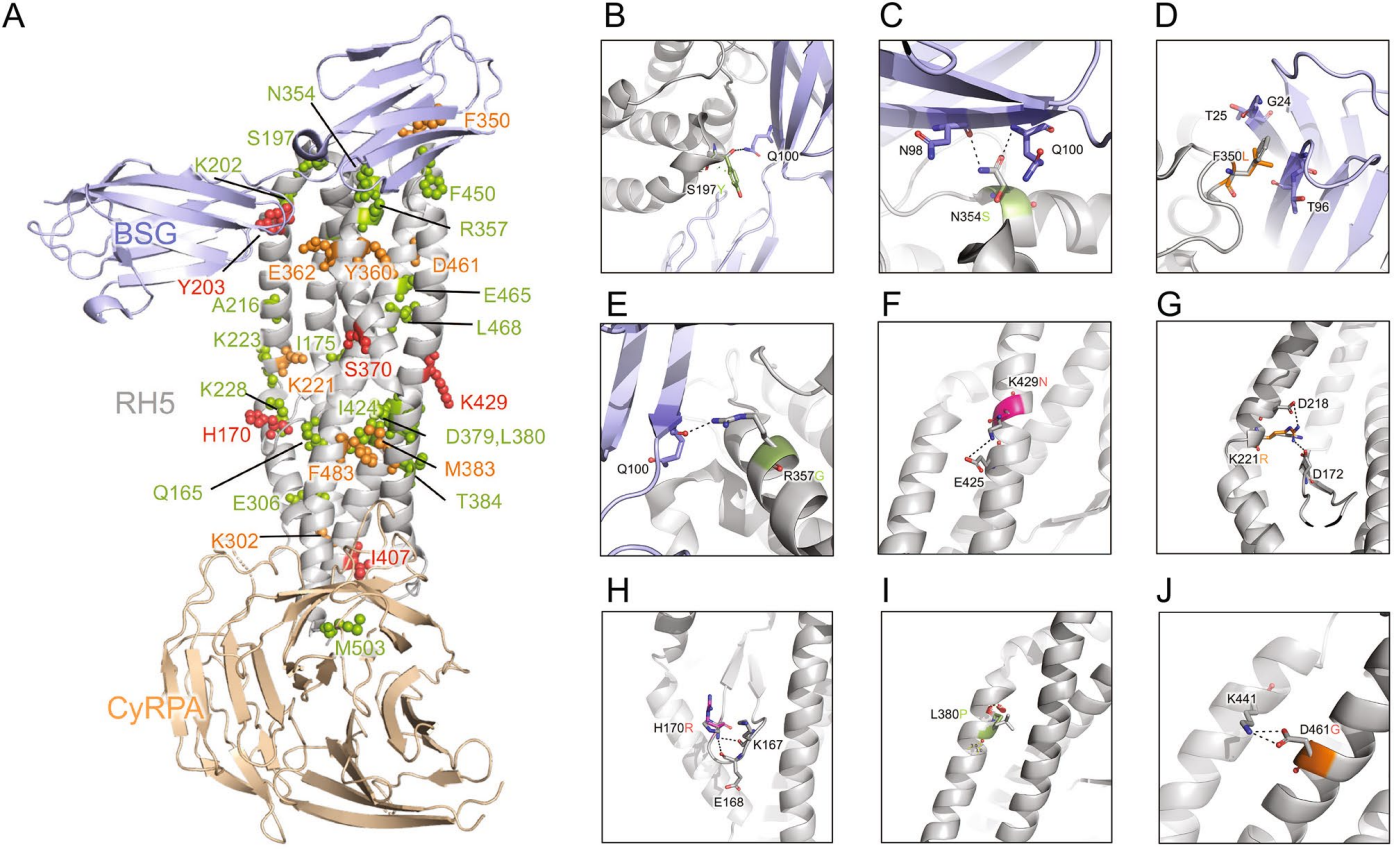
Structure-function predictions for novel SNPs identified in PfRh5 (**A**) Location of SNPs in the BSG–RH5–CyRPA complex. The complex was built by superposing the RH5 of RH5–BSG complex (PDB id: 4U0Q) and RH5–CyRPA complex (PDB id: 6MPV). BSG, RH5 and CyRPA are shown by light blue, grey and light orange, respectively. (**B**–**J**) Small panels highlight the predicted effect of selected SNPs, the black dotted lines represent hydrogen bonds or salt bridges. From top-left to bottom-right: SNPs that potentially impact binding of PfRh5 and BSG (**B**–**E**); S197Y, N354S, F350L, R357G), SNPs that potentially alter the structure of PfRh5 (**F**–**J**; K429N, D461G, L380P, H170R, K221R). SNPs reaching significance above the 1% discovery threshold are color coded according to frequency in individual samples with low frequency < 2% (green), intermediate frequency 2–25% (orange), high frequency > 25% (red).

We used structure-based approaches to thread the identified SNPs into the crystal structure of PfRh5 in complex with its receptor Basigin and binding partner PfCyRPA (Fig. 4A). The complex was built by superposing the RH5 of RH5–BSG complex (PDB id: 4U0Q) and RH5–CyRPA complex (PDB id: 6MPV) The structural threading analysis revealed four groups of SNPs with different predicted functional outcomes. One of the groups includes PfRh5-associated polymorphisms predicted to alter binding to the Basigin receptor (e.g. C203Y, S197Y, N354S, F350L, R357G, E362A/D) (Fig. 4B–E). Both S197 and R357 form a hydrogen bond with the Q100 of Basigin. Mutations at these residues such as S197Y and R357G could disrupt this hydrogen bond and decrease the binding affinity. Whereas N354 forms two hydrogen bonds with both N98 and Q100 of Basigin, the N354S mutant potentially decreases the interaction. The F350L mutation is present near the BSG–RH5 interface and may reduce the affinity. To estimate the binding affinities for these SNPs located in the interface, we used FoldX to calculate the interaction energy changes between WT RH5–BSG complex and mutated RH5–BSG complexes (Supplementary Table 2). The results showed that C203Y dramatically enhanced the binding affinity, and N347Y showed a moderate increase the interaction. While S197Y showed minor effect to the binding affinity, F350L, N354S, E362A and R357G were predicted to decrease the binding. Another subset of SNPs was predicted to impact PfRh5 binding to its partner PfCyRPA (e.g. M503T) (Fig. 4A). While the majority of the identified SNPs were predicted to partially alter the structure of PfRh5 (Fig. 4F–J), there was a particular set of SNPs for which no structural data was available, but occurring at high frequencies within the sampled populations. Interestingly, our analysis also revealed a group of SNPs occurring under known mapped binding epitopes for inhibitory antibodies (S197Y, K202R, R357G, and E362A). Of these, only S197Y has been shown to result in immune escape in parasites harboring this mutation^25^. These remaining three SNPs are novel, with K202R having been reported in our previous study^20^, and mutations have previously been described at amino acid position E362, but with a different amino acid change (E362D)^18^ compared to E362A reported here.

## Discussion

We sought to provide a thorough analysis of the genetic diversity present in the *P. falciparum* gene PfRh5 in Kédougou, Senegal, to further assess if this gene is an adequate target for a highly effective second-generation malaria vaccine. Previous research has shown PfRh5 to be relatively conserved, with 43 non-synonymous SNPs reported in published and unpublished data^18–23^. In our study, we found that PfRh5 exhibits more abundant genetic diversity in field isolates than previously thought, including the discovery of 74 novel SNPs. Implementing deep amplicon NGS on our 189 samples and setting a sensitive discovery threshold of 1% prevalence allowed us to discover these previously undescribed SNPs.

As Kédougou is a region with significant polyclonality, ranging from 1 to 15 genotypes per patient and an average complexity of infection (COI) of 4.59, it was important to assess not just the presence of SNPs per individual, but the frequency of these SNPs in the complex mixture of parasite genomes in each sample. To this end, we assessed the “frequency” of each SNP by assessing the percentage of variant reads in the total reads mapped to a given position in PfRh5. This value allows us to calculate quantitatively the number of variant reads in the total, taking into account polygenomic infections and also varying parasitemias of each genotype in the infected individual. Using this approach, we were able to classify the SNPs into those that were high (> 25%), intermediate (2–25%), and low (< 2%) in samples containing these SNPs. While most SNPs that were among the most prevalent in the population were also high frequency in individual positive samples, there were some exceptions whereby a single sample contained a SNP (low population prevalence), yet that SNP was present in a high frequency of the reads. Likewise, some SNPs were present in multiple samples resulting in a higher population prevalence, but were always at a low frequency in the individual sample. These distinctions will be important to explore through functional assays assessing the fitness of individual SNPs when compared to wild-type. It is also possible that these low frequency SNPs may also represent deleterious mutations that have not yet undergone purifying selection in the host; however, it has been observed that purifying selection is highly efficient in the malaria parasite life cycle^27^ .This division into frequency gives a more detailed picture of common and rare SNPs as it incorporates the complexity of the highly mixed infections. Describing SNPs in terms of frequency is also helpful in prioritizing SNPs for downstream follow-up.

Our site-specific analysis identified varying trends across sites that can be partially explained by their unique epidemiologies, locations, and capacities. Camp Militaire is a large military clinic that has a very large catchment area. As such, it is not surprising that this site included more samples through passive case detection and identified more individual SNPs than the others. Bantaco presents an interesting situation where over 90% of samples contained at least one SNP, about 10% higher than the other sites. Bantaco is a village that contains one of the largest traditional gold mining sites in the region. This mining site attracts workers from a variety of locations, including across the borders of Mali, Gambia, Guinea-Bissau, and Guinea, as well as countries that do not share a border such as Togo and Côte d’Ivoire, among others. This unique ecology facilitates the import of unique parasite variants from the West African sub-region, resulting in the mixing of parasite populations, naturally increasing the population-level genetic diversity. The Bantaco and Bandafassi sites are more isolated than the other sites, which sit near roads and can service patients that are not necessarily currently living near the clinic. We expected to see more allele sharing across Mako, Camp Militaire, Dalaba, and Tomboronko given their proximity to roads and thus the higher likelihood of patient populations coming from similar areas. This was observed with certain SNPs, such as D249G and H170R being found in Dalaba, Mako, and Camp Militaire. C203Y was the only SNP found across all sites, which is expected given our previous documentation of its high prevalence in the region^20^, in other regions of Senegal^21^, and globally^18,24^. Overall, the sample size from each clinic does not truly permit quantitative comparisons of genetic diversity between sites; however some of these trends are interesting and would warrant further investigation in prospective targeted studies.

While characterization of genetic diversity in vaccine candidate antigens is important, ultimately functional studies will be needed to appreciate the role of specific polymorphisms on biological functions like receptor binding, PfRh5–PfCyRPA–PfRipr (RCR) complex formation, and immune evasion. To this end, to generate testable hypotheses as to which SNPs may be important to prioritize for future functional studies, crystal structure threading of the observed SNPs allowed us to uncover potential functional impacts on the PfRh5 complex, its binding partner PfCyRPA, and the associated erythrocyte receptor Basigin. Most of the SNPs are predicted to result in minor changes to the protein’s structure. Importantly, our analysis found 4 SNPs (S197Y, K202R, R357G, and E362A) that occur beneath well-defined epitopes bound by neutralizing human monoclonal antibodies from PfRh5 vaccinated individuals^25^. Of these, only S197Y has been previously described and was found to aid parasites in evading the immune response^25^. Lack of crystal structure data for a subset of our observed SNPs prevented structural threading, thus we cannot predict the potential functional impact of these SNPs.

Our study has some limitations that are important to note. First, the sample sizes across sites are not equivalent due to enrollment through passive case detection of febrile patients who come to the respective clinic and give informed consent to participate in the study after testing positive for *P. falciparum*. This sampling strategy does not represent a true cross-sectional population prevalence as might be achieved using systematic cross sectional sampling or genotyping from discarded diagnostic materials. This could potentially bias analysis of genetic diversity towards parasite strains that may cause more prominent clinical disease that influences the patient to seek care. Future work can address this by sampling across the clinical presentation spectrum, including active surveillance of asymptomatic cases. Further, while our sequencing approach has advantages for deep coverage and detection or rare SNPs, it has its caveats as well. Due to the short reads and the high complexity of infection in this population, we are unable to resolve individual parasite haplotypes which would allow us to perform population genetic analyses comparable to other studies that have focused on monogenomic infections and employed capillary sequencing^22, 23^.

While our structural modeling can imply potential functional outcomes of the SNPs identified in PfRh5, further experimental work is needed to fully assess the potential functional impacts of these SNPs, in binding to the Basigin receptor, binding to PfRh5 binding partners, and in immune evasion in the presence of naturally acquired and vaccine-induced immune responses. This study will inform downstream biochemical and functional genetic approaches to evaluate the role of each SNP in receptor binding, invasion, and immune evasion.

## Methods

### Study sites

This study was conducted with ethical approval from the National Ethics Committee of Senegal (CNERS) and the Institutional Review Board of the Yale School of Public Health. All research was performed in accordance with relevant guidelines and regulations, and informed consent was obtained from all participants and/or their legal guardians.

Samples used in this study were collected as part of ongoing surveillance conducted by Institut Pasteur de Dakar investigating causes of febrile illness. Patients were recruited from six health posts across Kédougou, Senegal: Bantaco, Mako, Camp Militaire, Bandafassi, Dalaba, and Tomboronkoto. The only eligibility criteria for the main study was the presence of a fever (temperature greater than or equal to 38 °C) and/or a fever in the past 24 h. Study clinical staff assessed eligibility and obtained informed consent from patients who tested positive for *P. falciparum* on a Pf-specific HRP2/3 rapid diagnostic test (RDT). After a venous blood draw of 5ml in an EDTA vacutainer was obtained from consenting, enrolled patients, samples transported at room temperature to the field lab for processing; no more than 6 h between draw and processing. Thin and thick blood smears were made for each sample to confirm infection with only *P. falciparum* by microscopy.

### DNA extraction, amplification, and sequencing

DNA was extracted from dried blood spots (DBS) using QIAmp DNA Blood Mini Kit according to manufacturer’s instructions. PfRh5 exon 2 was PCR amplified using previously described primers^21^ and high fidelity polymerase with proofreading capability. PCR amplicon size and purity were confirmed on an agarose gel prior to Next Generation Sequencing (NGS). PCR amplicons for PfRh5 exon 2 were bead-purified (Omega) and quantified by Qubit and adjusted to equivalent concentration. Library prep was performed with Nextera XT using unique dual indexes (UDIs). After library prep, samples were again bead-purified and quantified by qPCR using Roche KAPA Library Quantification Kit. All samples were normalized to a concentration of 4 nM. After this normalization, quantified and normalized libraries were pooled into 8-sub pools. These 8 sub-pools were bead-purified and quantity was measured by KAPA qPCR. The 8 sub-pools were normalized and combined in equal parts to form one final pool. This final pool was sent to the Yale Center for Genome Analysis (YCGA) for sequencing on an Illumina NovaSeq 6000 platform with targeted coverage of 500,000 reads per sample.

De-multiplexed forward and reverse sequencing reads were obtained for each sample. These reads were imported to Geneious individually then paired. This was followed by trimming using the Geneious plugin BBDuk. A minimum Quality Score (Q) of 30 was set for the trimming with a minimum length of 75 base pairs, as we were expecting reads around 100 base pairs. The trimmed sequences were mapped against a 3D7 reference genome (PF3D7_ 0424100) that had been annotated with all known synonymous and non-synonymous mutations. The mapping was set for two iterations and coverage criteria was set at 1000 reads. The criteria for SNP calling was set to a minimum frequency of 0.01 (1%) and 1000 read coverage. Sequence data and SNP analysis was performed by at least 3 individuals for each sample.

As a control for PCR and sequencing fidelity, we sequenced PfRh5 amplified from 3D7 genomic DNA alongside each batch of samples. This control was to ensure that rare SNPs are not merely the result of PCR or sequencing errors when applying a SNP discovery threshold of 1%.

### Complexity of infection

Complexity of infection (COI) for each sample was determined through genotyping the merozoite surface proteins (MSP) with a nested PCR on DNA extracted from whole blood, as described by Snounou^28^.

### Structure modeling of SNPs

The structures of BSG–RH5 and CyRPA–RH5 were downloaded from the Protein Data Bank (PDB, https://www.rcsb.org/) under accession number 4U0Q and 6MPV, respectively. The BSG–RH5–CyRPA complex was built by superposing the RH5 in the BSG–RH5 and CyRPA–RH5 complexes. Individual FASTA files containing amino acid sequences of RH5 and individual novel SNPs were generated. These amino acid sequence files were threaded through the crystal structure. Pymol version 2.3.2 was used to predict the effect and to plot the structural location of each SNP^29^. The structural effect of the mutant versions of the protein were evaluated in terms of biochemical properties such as hydrogen bonding patterns, steric interactions, and predicted binding affinity between the mutant version of the protein and the Basigin receptor The binding energy alternation for SNPs and BSG were predicted by FoldX version 5.0^30^.

### Statistical analysis

Summary statistics of patient demographics were performed and comparisons were made across the six study sites. Sex ratios were compared using the Chi-squared test across sites. Comparisons of average age and COI across sites were compared using One-way ANOVA. P-values $\le$ 0.05 are considered significant.

## Supplementary Information

Supplementary Information.

Supplementary Table S1.

## Data Availability

Sequencing Reads associated with this study have been deposited in the NCBI SRA with the BioProject Accession: PRJNA811159.
