# Supplementary material for: Structure-guided insights into potential function of novel genetic variants in the malaria vaccine candidate PfRh5
Source: Sci Rep. 2022 Nov 12;12:19403. doi: 10.1038/s41598-022-23929-9 (PMC9653458; doi:10.1038/s41598-022-23929-9)
Supplement: Supplementary file 1 — Supplementary Information. [file 41598_2022_23929_MOESM1_ESM.pdf]

Supplementary Information for

Structure-guided insights into potential function of novel genetic variants in the malaria vaccine candidate PfRh5

Khadidiatou Mangou, Adam J. Moore, Laty Gaye Thiam, Aboubacar Ba, Alessandra Orfanó, Ife Desamours, Duncan Ndungu Ndegwa, Justin Goodwin, Yicheng Guo, Zizhang Sheng, Saurabh D. Patel, Fatoumata Diallo, Seynabou Diouf Sene, Mariama Nicole Pouye, Awa Thioub Faye, Alassane Thiam, Vanessa Nunez, Cheikh Tidiane Diagne, Bacary Djilocalisse Sadio, Lawrence Shapiro, Ousmane Faye, Alassane Mbengue, Amy K. Bei

Amy Kristine Bei
E-mail: amy.bei@yale.edu

This PDF file includes:

Fig. S1
Tables S1 to S2

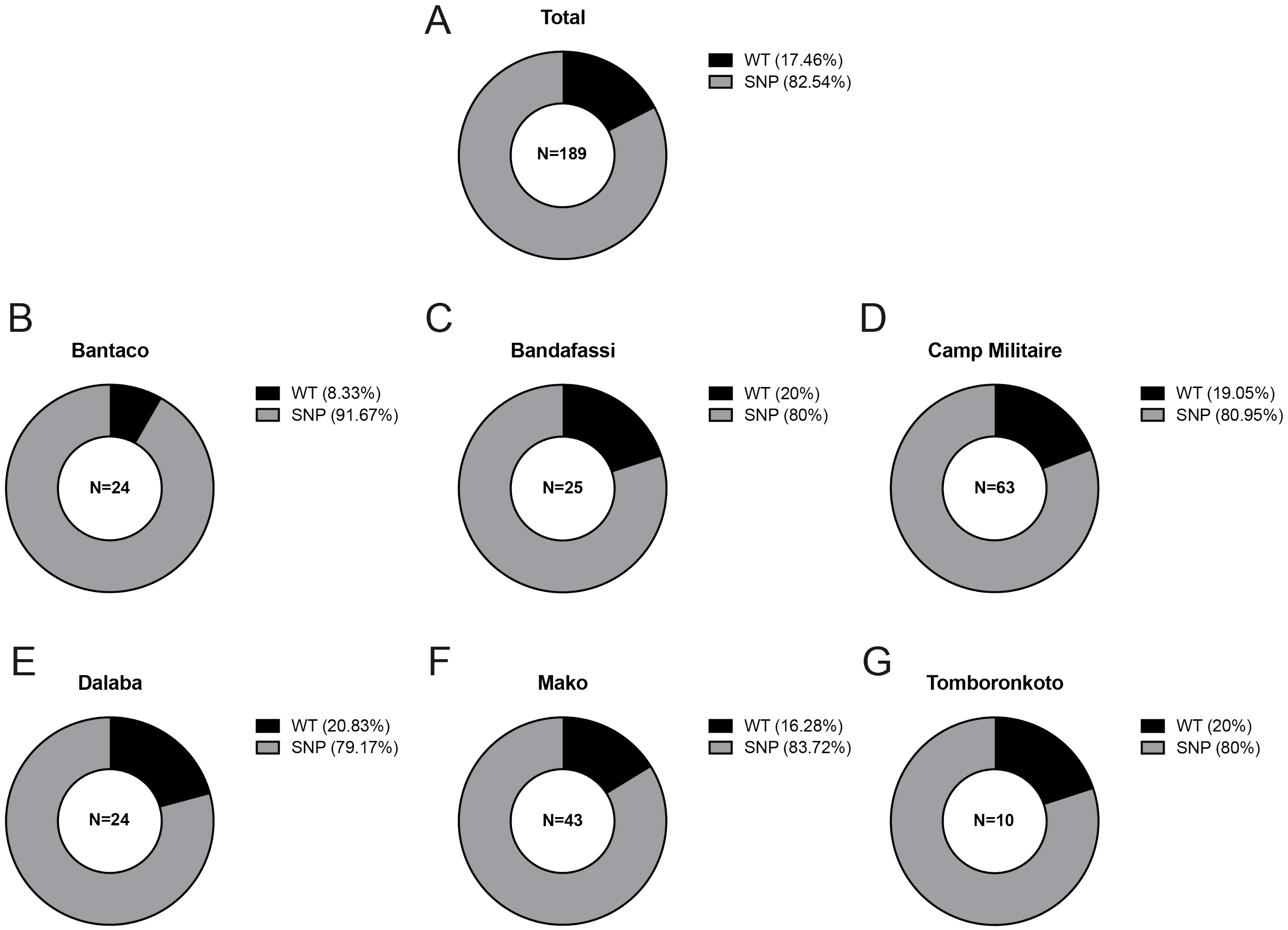

Fig. S1. Population prevalence of wild-type and mutant PfRh5 alleles

The prevalence of wild-type (3D7-allele) and mutant PfRh5 alleles (any mutation) was calculated as the percentage of isolates containing one or more SNPs at the discovery threshold (>1% variant frequency) relative to the total sample population (A) or site-specific sample populations (B-G) in Kédougou.

			Coverage at SNP Position			Variant Reads			
Sample ID	Site	SNPs	Mean	Minimum	Maximum	Mean	Minimum	Maximum	Variant Read Frequency (%)
320290	BF	C203Y	39451	39313	39727	39434	39299	39703	100
320294	CM	N66D	7478	7478	7478	974	974	974	13
		C203Y	28800	28724	28952	28486	28486	28712	99
		D461G	7977	7977	7977	124	124	124	2
320301	BC	C203Y	57851	34384	69585	46498	352	69571	67
320322	DB	C203Y	21038	21010	21094	3047	3040	3060	14
		T216A	22812	22767	22901	236	235	238	1
		N354S	10230	10201	10289	131	130	133	1
320323	MK	V371I	12117	12097	12157	3237	3232	3247	27
320324	CM	C203Y	28738	28654	28907	28730	28648	28894	100
		K302R	17183	17002	17545	474	469	484	3
		I420T	5979	5973	5990	69	69	69	1
320325	CM	C203Y	26332	26264	26469	26261	26257	26456	100
320328	CM	N75D	5516	5480	5588	80	76	85	1
		C203Y	23931	19379	26329	17608	422	26321	67
		F350L	10595	2205	19379	2977	24	8486	29
		I407V	14892	10200	19583	4459	431	8486	43
320336	MK	H148D	4117	4108	4135	1244	1241	1250	30
		C203Y	22159	22095	22286	22152	22089	22278	100
		V371I	9878	9871	9893	4349	4346	4355	44
320343	TM	C203Y	25800	25729	25943	25711	25711	25922	100
320344	MK	C203Y	27466	27318	27761	27393	27314	27551	100
320445	MK	C203Y	20422	20368	20531	4707	4681	4760	23
320346	TM	C203Y	20769	20727	20852	2134	2122	2157	10
		I407V	5461	5453	5476	4043	4038	4053	74
320364	CM	C203Y	12199	12165	12267	7089	7064	7140	58
320393	MK	C203Y	8159	4279	15885	5336	64	15877	34
		R357G	13978	10066	15983	10675	177	15972	67
320398	DB	C203Y	36943	10066	68048	2834	177	5987	6
		I407V	19737	19151	20323	17994	17452	18536	91
320406	MK	C203Y	50844	50725	51081	8625	8575	8724	17
320558	BC	C203Y	50630	45408	53241	50611	45385	53224	100
		I407V	22662	22662	22662	8945	8945	8945	39
		K429N	17974	17974	17974	10608	10608	10608	59
320564	DB	C203Y	26270	25630	27549	25607	25607	27521	100
320576	BC	C203Y	36486	36485	36489	36476	36476	36477	100
320602	CM	C203Y	136955	133695	138585	138439	133505	138439	100
320603	CM	C203Y	88326	87968	88505	88209	87837	88395	100
320609	CM	C203Y	46277	46277	46277	46262	46262	46262	100
320254	BF	Q165R	2294	2047	2429	32	32	32	1
		I176M	3202	3161	3284	98	97	101	3
		C203Y	14071	13848	14516	14067	13844	14512	100
		S370P	2412	1767	2735	64	63	65	3
320258	BC	C203Y	89125	88948	89479	89088	88918	89427	100
		D218G	84045	83082	84526	923	901	968	1
		I424V	12551	12346	12961	147	141	159	1
320278	CM	N70D	5284	729	7561	731	729	734	40
		N90D	4549	78	6784	78	77	78	34
		F99S	3910	145	5792	143	139	145	34
		Y147C	4101	94	6104	97	94	102	37
		L152S	6403	462	9374	475	462	500	39
		C203Y	99233	99184	99257	98567	97333	99184	99
		N297S	39010	1601	57715	1629	1601	1684	37
		F450L	6382	375	9385	388	375	414	39
320303	MK	C203Y	44041	43879	44364	44030	43866	44344	100
		E465D	7491	7483	7506	93	92	94	1
320319	CM	K228E	13036	12930	13249	205	202	210	2
320334	TM	K58R	6028	6023	6031	86	85	88	1
		K221R	20862	20787	21013	414	413	417	2
		I442V	2439	2435	2448	30	30	31	1
320340	BF	C203Y	40284	40126	40600	40271	40115	40584	100
		E322G	2817	2810	2831	33	33	33	1
		L468S	8183	8183	8183	86	86	86	1
320344	MK	C203Y	27466	30924	31346	27393	3995	4109	100
320345	MK	C230Y	20422	6498	6563	4707	5260	5260	23
320366	TM	C203Y	31065	18429	19548	4033	258	305	13
		I407V	6520	145443	155078	5260	145385	155043	54
320367	DB	E63D	19175	34764	35076	274	620	643	1
		C203Y	151866	4330	4331	151824	48	49	100
		L380P	34868	11819	57092	628	124	56764	2
320378	BF	Y82C	4330	28629	61411	49	670	61043	1
		N159D	26942	22453	28629	19008	378	670	34
		C203Y	49044	25311	25501	39492	25136	25321	67
		D249N	25541	34550	34794	524	34542	34783	2
320387	BC	C203Y	25374	4839	5254	25198	58	66	99
320392	BF	C203Y	34631	45942	46762	34622	45938	46750	100
320395	DB	K58E	4977	20010	20473	61	399	431	1
		C203Y	46215	5389	8743	46209	5382	5526	100
		K247E	20164	8900	8900	410	89	96	2
		K429N	6557	5685	5826	5430	64	66	87
		M503T	8900	62998	64318	93	31524	32286	1
320397	DB	K140R	5732	13367	14449	65	8160	8772	1
		C203Y	63438	26518	26689	31778	16667	26502	50
		I407V	13728	12018	12151	8364	133	135	61
320400	CM	C203Y	26575	40506	40679	23224	586	596	87
320404	CM	D52G	12062	39244	39607	134	421	434	1
		C203Y	40564	3430	3437	589	60	60	1
		Y217C	39365	32906	33185	425	32857	33133	1
320405	CM	N117H	3432	25234	25413	60	269	276	2
		C203Y	32999	105287	105729	32949	63618	63840	100
		K223R	25294	61742	84193	271	41767	57341	1
		D269N	105434	10569	10602	63692	202	202	60
320431	CM	C203Y	76709	28656	28928	52150	28648	28916	68
320433	BC	K58R	10580	38438	39756	202	402	426	2
		C203Y	28747	58304	62442	28740	58270	62407	100
		N284D	38877	17598	17691	410	177	182	1
320434	BC	C203Y	59683	33274	33545	59660	33268	33537	100
320435	BC	E48G	17660	6981	7000	179	89	89	1
		C203Y	33364	34668	35000	33358	25317	25592	100
		K511N	6991	4504	4610	89	49	52	1
320440	MK	C203Y	34779	3790	3814	25409	50	50	73
320443	CM	K77E	4539	3883	3906	50	1589	1594	1
		D127G	3798	3947	3966	50	1604	1610	1
		Y147H	3891	27043	27305	1591	27033	27288	41
		H148D	3953	22017	22438	1606	264	283	41
		C203Y	27130	19202	20089	27121	19201	20088	100
		D249G	22157	5669	5684	270	130	130	1
320444	CM	C203Y	19498	45162	45529	19497	45158	45515	100
320445	CM	G119D	5674	91239	92678	130	5032	5136	2
		C203Y	45284	104701	105895	45278	3308	3360	100
		D255G	91719	13092	13245	5067	258	265	6
		D276G	105099	16931	18458	3325	198	267	3
		Y360H	13143	16931	17007	260	198	202	2
320446	CM	K45R	17838	35751	35800	239	30531	30568	1
		G336S	16969	40062	40476	200	40054	40561	1
320448	DB	C203Y	35767	34169	34412	30543	13243	13393	85
320455	DB	C203Y	40200	9863	9919	40223	6178	6217	100
320464	BC	C203Y	34250	30002	30252	13293	29910	30157	39
		I407V	9882	29199	29475	6191	7420	7539	63
320465	BC	C203Y	30085	54244	54592	29992	54217	54573	100
320466	BC	C203Y	29291	22387	22743	7460	9969	10210	25
320467	BC	C203Y	54476	22534	22705	54454	22523	22692	100
320481	MK	C203Y	22506	52156	52722	10049	52138	52716	45
320484	CM	C203Y	22591	19165	19165	22579	1056	1056	100
320485	CM	C203Y	52533	26657	26967	52523	26360	26663	100
		V371I	19165	61141	61580	1056	3614	3753	6
320486	CM	C203Y	26760	46099	61758	26461	733	859	99
320488	CM	C203Y	61287	20459	57213	3660	302	57211	6
		S251Y	56538	57213	160757	817	6430	57211	1
320489	DB	S46P	40324	21218	129837	19348	276	6430	34
		C203Y	115936	21218	21218	23485	276	276	36
		S257P	62519	50991	51447	2353	47994	48434	2
		D379G	21218	8695	8744	276	679	8695	1
320490	DB	C203Y	51143	37719	38121	48141	37707	38104	94
		I407V	8711	23108	23808	3353	234	291	39
320491	DB	C203Y	37853	5048	5061	37839	51	51	100
		D249G	23341	9236	9327	253	256	259	1
		R423G	5052	10939	11010	51	790	790	1
320495	DB	K58E	9266	35963	36216	257	17083	17249	3
		H170R	10963	11425	11505	790	211	213	7
		C203Y	36047	30449	30635	17138	30438	60621	48
		T384A	11452	5127	5143	212	5123	5138	2
320538	CM	C203Y	30511	5199	5317	30503	69	76	100
		V371I	5132	4423	4513	5128	113	118	100
320549	CM	N66S	5278	8907	9448	74	116	122	1
		F483L	4468	3339	3357	116	426	432	3
320575	BC	E50G	9087	58658	59275	118	15207	15557	1
		H148D	3345	4781	5622	428	106	114	13
		C203Y	58864	7534	7623	15324	78	82	26
		T309K	5061	10135	10178	109	2612	2622	2
		C329R	7564	71508	75173	79	987	1011	1
		I407V	10149	94327	97664	2615	2158	27447	26
320579	TM	K202R	73951	23352	94327	995	1849	9452	1
		C203Y	95979	22275	23352	18966	2880	9452	20
		S251F	64806	17653	23629	4486	2880	7090	15
		V371I	22707	17653	18542	7115	7090	7474	31
		I407V	21258	61210	62388	4317	10521	10913	22
		I410M	18098	68738	71636	7282	68656	71563	40
320580	TM	C203Y	61603	101521	103544	10652	101489	103494	17
320581	TM	C203Y	70670	93436	96695	70594	93383	96620	100
320582	TM	C203Y	102195	212867	248416	102157	212721	248416	100
320599	CM	C203Y	94522	15457	16558	94462	282	284	100
320600	CM	C203Y	236566	63040	63735	236486	765	767	100
		E322G	15824	30648	30761	283	325	340	2
		M383R	63503	122739	126244	766	122528	126061	1
320604	CM	I175M	30686	3558	56751	330	36	56734	1
		C203Y	125076	47973	49589	124883	47950	49573	100
320628	MK	C203Y	38982	21753	22036	37790	21753	22035	67
320630	MK	C203Y	49050	34384	35153	49032	352	361	100
320631	MK	C203Y	21847	8819	8866	21847	101	103	100
320719	BC	L380P	34897	32932	33257	358	32932	33252	1
320720	BC	D65G	8835	8114	8223	102	224	237	1
		C203Y	33040	20634	47078	33039	290	47056	100
		E362A	8150	21990	47078	228	320	47056	3
320784	MK	P43L	36662	47626	47626	15976	47594	47594	34
		S46G	34534	34694	35008	23688	34678	34986	51
		C203Y	47626	10309	10333	47594	132	159	100
320787	MK	C203Y	34799	3402	3423	34781	38	38	100
		V371I	10317	11123	11270	150	216	219	1
320789	MK	V142I	3409	50199	50644	38	24298	24597	1
320790	MK	H170R	11172	159237	159538	217	1663	1673	2
		C203Y	50347	1029	1052	24398	12	1052	48
		T267A	159337	3984	4050	1666	3076	3133	1
320620	BC	K51E	1037	1459	1495	359	1459	1493	34
		C203Y	4006	1414	1431	3095	1414	1431	77
320633	MK	C203Y	1471	1979	2011	1470	720	733	100
320636	DB	C203Y	1420	5626	5823	1420	5617	5814	100
320639	MK	C203Y	1990	22904	25915	724	12197	13145	36
320658		C203Y	5692	5829	6449	5716	73	76	100
320660	BF	E306G	24911	6990	7357	12829	3927	4105	52
		I407V	6242	26460	26694	75	26441	26685	1
		C203Y	7235	37042	84267	4046	36980	84206	56
320661	BF	C203Y	26616	15263	15825	26563	5043	5260	100
320302	BC	C203Y	63867	8519	9012	63796	8504	8997	100
320358	BC	C203Y	15544	13457	13777	5152	7764	7861	33
320375	CM	C203Y	8848	10129	10385	8833	107	110	100
320381	CM	K223R	13670	55187	74470	7829	47294	64258	57
		C203Y	10214	14360	15177	108	1190	1241	1
320384	CM	I407V	68042	5664	5727	58603	5662	5723	86
		C203Y	14905	3927	4106	1224	3921	4100	8
320403	MK	C203Y	5685	10263	10979	5682	130	143	100
320407	CM	Y252H	4046	4914	5128	4040	4904	5118	100
		C203Y	10502	1155	1332	134	19	31	1
320408	CM	Y252H	5057	1243	1338	5047	738	785	100
		I407V	1234	5151	5407	27	5144	5392	2
		C203Y	1306	19847	20829	769	19813	20798	59
320411	DB	C203Y	5236	5171	5418	5227	5171	5416	100
320412	DB	I407V	20502	12359	18914	20470	129	279	100
320414	BC	Y252H	5253	3629	3629	5253	279	279	100
320416	BC	M330T	16729	1614	16147	195	6080	6508	1
		C203Y	3629	4510	14865	279	1329	4510	8
320468	MK	N289D	10931	4184	4510	6365	1168	2117	27
		V371I	9564	4065	4211	2647	2053	2117	43
		I407V	4302	2255	2277	1507	2253	2275	35
		C203Y	4138	4659	4659	2085	4638	4638	50
320469	MK	C203Y	2270	1554	1580	2268	1554	1579	100
320472	MK	C203Y	4659	3309	3379	4638	154	158	100
320473	MK	C203Y	1571	4710	4805	1571	341	345	100
320478	MK	C203Y	3344	4552	4552	156	50	50	5
320502	CM	K223R	4758	12454	15556	343	5814	7269	7
		C203Y	4552	8622	8860	50	8611	8847	1
320504	CM	C203Y	14522	19905	20529	6784	19873	20501	47
320510	MK	C203Y	8781	7305	7417	8768	7288	7400	100
320511	MK	C203Y	20321	43919	51351	20292	43826	51249	100
320513	MK	C203Y	7380	35463	35463	7363	395	395	100
320517	MK	K223R	48874	3658	3804	48775	3652	3799	100
		C203Y	35463	19573	20738	395	19529	20702	1
320519	MK	C203Y	3755	61434	78462	3750	61278	78301	100
320524	CM	C203Y	20350	4630	4716	20311	3225	3283	100
320525	CM	C203Y	72786	3349	3386	72627	3345	3382	100
320541	CM	C203Y	4687	19687	22763	3264	19637	22718	70
320545	BC	C203Y	3374	32178	37845	3370	32119	37771	100
320548	CM	C203Y	21738	8750	18539	21691	211	9134	100
320550	CM	E306G	35956	9143	9143	35887	9134	9134	100
		I407V	12144	3699	3770	6026	3690	3761	67
		C203Y	9143	1121	1191	9134	1117	1191	100
320557	CM	I407V	3746	6005	6282	3737	5996	6275	100
		C203Y	1168	9087	9777	1165	9082	9775	100
320560	DB	C203Y	6190	21706	23162	6182	21673	23139	100
320562	DB	C203Y	9547	23156	24449	9544	23124	24427	100
320563	DB	C203Y	22677	12057	14213	22650	127	143	100
320574	BC	E306G	24018	71786	85923	23993	45518	71729	100
		C203Y	13135	25073	27980	135	3599	3843	1
320577	BC	C203Y	76498	7235	7996	62992	74	1095	84
320583	MK	V371I	27011	9201	9206	3762	9003	9005	14
		C203Y	7742	7115	7610	422	7102	7600	5
320585	MK	C203Y	9203	13020	14474	9004	13009	14465	98
320586	MK	C203Y	7445	19096	22021	7434	18699	21579	100
320587	MK	C203Y	13989	22775	23129	13980	17388	17526	100
320589	MK	C203Y	21046	4681	80064	20619	4668	80010	98
320595	DB	H148D	23011	52076	80064	17480	52012	80010	76
320611	CM	C203Y	30060	1518	1541	30037	1514	1536	100
		C203Y	66070	7933	8344	66011	7922	8336	100
320619	TM	C203Y	1533	2467	2786	1529	885	2467	100
320664	BF	I407V	8207	5806	5924	8198	3401	3484	100
		C203Y	2656	2475	2567	1919	31	37	71
320665	BF	E306G	5885	8070	8112	3456	8065	8108	59
		C203Y	2521	8592	8938	34	8245	8578	1
320666	BF	C203Y	8098	4329	4446	8094	4321	4442	100
320667	BF	I407V	8823	1684	1704	8467	1309	1322	96
320668	BF	C203Y	4407	1973	2007	4402	1566	1583	100
320669	BF	C203Y	1697	2218	2266	1318	2215	2264	78
320671	BF	C203Y	1996	78095	399314	1577	50109	261171	79
320674	BF	C203Y	2250	6439	63145	2248	2519	20920	100
320675	BF	T299K	238705	19786	69726	155640	3721	11884	65
		V371I	34792	2568	2578	11720	64	65	36
		C203Y	44756	6117	6191	7803	3928	3966	18
320676	BF	C203Y	2573	2076	2114	65	1588	1624	3
320692	BF	C203Y	6166	1341	1347	3953	1339	1347	64
320695	CM	C203Y	2101	5833	5920	1612	5826	5909	77
320696	CM	C203Y	1345	11196	12019	1344	3365	3639	100
320698	CM	C203Y	5862	16140	16866	5854	11963	12454	100
320701	CM	N248Y	11745	3308	3541	3548	3302	3537	30
		C203Y	16382	4165	4326	12127	4163	4326	74
320707	CM	C203Y	3463	7016	7415	3459	4410	4629	100
320710	CM	C203Y	4272	1891	5812	4272	603	5809	100
320637	DB	C203Y	7282	1697	1768	4556	1694	1766	63
320651	MK	I407V	4442	4252	5624	4009	1746	5615	77
		C203Y	1733	27318	27761	1730	27314	27551	100
320656	BF	C203Y	4709	20368	20531	3036	4681	4760	61

Table S1. Description of the SNPs identified in patient samples from Kédougou

Table S1 is included as a supplemental file (.xls). Sampling sites are abbreviated as follows: Bandafassi (BF), Bantaco (BC), Camp Militaire (CM), Dalaba (DB), Mako (MK) and Tomboronkoto (TM). For each isolate, non-synonymous amino acid substitutions are indicated along with their respective statistics (Coverage at SNP position, Variant Read coverage, and Variant Read frequency).

SNPs	WT(Kcal/mol)	Mutated(Kcal/mol)	ΔΔG(Kcal/mol)
Y358F	-20.36	-20.54	-0.17
C203Y	-18.25	-20.84	-2.58
I204K	-20.15	-19.47	0.68
N347D	-20.14	-20.30	-0.16
N347Y	-20.21	-21.13	-0.92
S197Y	-20.27	-20.18	0.09
R357G	-20.75	-18.42	2.33
E362A	-20.51	-20.35	0.15
F350L	-20.63	-19.30	1.32
N354S	-20.82	-18.05	2.76

Table S2. Predicted binding energy alternations for BSG and RH5 variant proteins

Individual FASTA files with RH5 and individual novel SNP amino acid sequences were threaded through the crystal structure. The structural effect of the mutant versions of the protein were evaluated for predicted binding affinity between the mutant version of the RH5 protein and the Basigin receptor. The binding energy alternation for SNPs and BSG were predicted by FoldX version 5.0.. Predicted binding energies are shown for wild-type and mutant versions of the protein in Kcal/mol for each SNP. Changes between the two are shown as $\Delta\Delta G$(Kcal/mol). A negative $\Delta\Delta G$ indicates a predicted increase in binding and a positive $\Delta\Delta G$ indicates a predicted decrease in binding.
